# *FOXP3* Mutations and Instability as Determinants of Regulatory T-Cell Plasticity in Endocrine Autoimmunity

**DOI:** 10.3390/ijms27135778

**Published:** 2026-06-26

**Authors:** Manal A. Abbas

**Affiliations:** Department of Medical Laboratory Sciences, Faculty of Allied Medical Sciences, Al-Ahliyya Amman University, Amman 19111, Jordan; m.abbas@ammanu.edu.jo

**Keywords:** autoimmunity, endocrine, epigenetics, immune tolerance, FOXP3, regulatory T cells

## Abstract

Autoimmune endocrine diseases constitute a group of disorders characterized by immune-mediated destruction or dysfunction of hormone-producing glands. The pathogenesis of these diseases reflects a breakdown of immune tolerance in which regulatory T cells (Tregs) play a key role. The transcription factor forkhead box P3 (FOXP3) is a master regulator of Treg differentiation and suppressive function. Also, it is central to maintaining self-tolerance. Genetic mutations in *FOXP3*, including those responsible for immune dysregulation, polyendocrinopathy, enteropathy X-linked (IPEX) syndrome, highlight the critical role of FOXP3 in endocrine immune tolerance. Emerging evidence suggests that autoimmune endocrine disorders may reflect organ-specific destabilization of *FOXP3* expression rather than complete Treg deficiency. The reversibility or irreversible loss of *FOXP3* gene expression represents a key determinant of Treg plasticity and the persistence of autoimmune inflammation. This review proposes an integrated genetic–epigenetic model of *FOXP3* instability and examines how the endocrine microenvironment shapes Treg plasticity. Genetic or epigenetic alterations affecting *FOXP3* expression can impair Treg activity and precipitate endocrine organ-specific autoimmunity. Epigenetic mechanisms such as DNA methylation, histone modifications, and non-coding RNA-mediated regulation that modulate *FOXP3* transcriptional activity are discussed. From a translational perspective, the potential of FOXP3 as a biomarker for endocrine disease susceptibility and progression was summarized. Furthermore, therapeutic strategies employed for expanding or engineering functional FOXP3^+^ Tregs using antigen-specific vaccines, chimeric antigen receptors (CAR)-Tregs, gene therapy, or low-dose interleukin-2 (IL-2) were described.

## 1. Introduction

Autoimmune endocrine diseases represent a group of conditions in which the immune system targets hormone-producing glands. Autoimmune endocrine diseases are caused by autoreactive lymphocytes and autoantibodies directed against gland-specific antigens. This results in chronic inflammation and tissue destruction causing endocrine insufficiency or dysregulation. Examples include type 1 diabetes mellitus, autoimmune thyroid diseases (such as Hashimoto’s thyroiditis and Graves’ disease), autoimmune adrenalitis (Addison’s disease) [1,2,3], autoimmune hypophysitis [4] and the rare autoimmune hypothalamitis [5]. Additionally, patients may present with polyglandular autoimmune syndromes, where multiple endocrine glands are affected either simultaneously or sequentially [6].

The development of autoimmune endocrine diseases involves a complex interplay between genetic predisposition and environmental factors like infections or stress [6]. Genetic factors include variants in immune regulatory genes such as human leukocyte antigen (HLA) and forkhead box P3 (*FOXP3*) gene variants [6]. Understanding the pathogenesis of these disorders requires examining the mechanisms that maintain immune self-tolerance. In recognition of the importance of this field, the 2025 Nobel Prize in Physiology or Medicine was awarded for discoveries elucidating peripheral immune tolerance. This process is critical for preventing autoimmune disease. In 1995, regulatory T cells (Tregs), a subset of CD4^+^ T cells that suppress excessive or autoreactive immune responses, were first characterized [7]. Subsequent studies identified FOXP3 as a master transcription factor essential for Treg development and function. Dysregulation of FOXP3 or Treg function can precipitate autoimmune disease or, conversely, enable tumor immune evasion [8,9]. Experimental evidence has established a causal relationship between FOXP3, Tregs, and immune self-tolerance. Ectopic *FOXP3* expression or gene deletion demonstrated that it is both necessary and largely sufficient for Treg differentiation and suppressive function, whereas its absence causes severe systemic autoimmunity in mice. Genetic evidence from the scurfy mouse, which carries a *Foxp3* mutation, mirrors the phenotype of human *FOXP3* deficiency. Defective or absent *Foxp3* leads to a lack of functional Tregs resulting in uncontrolled autoreactive T cells and organ-specific autoimmunity [10]. These findings established the causal pathway linking *FOXP3*, Tregs, and self-tolerance.

Endocrine organs possess a unique microenvironment that may challenge the stability of Tregs and the maintenance of *FOXP3* expression. These metabolically active tissues are exposed to conditions that promote oxidative and inflammatory stress, causing destabilization of *FOXP3*. For example, pancreatic islets exhibit high levels of oxidative stress due to intense metabolic activity causing reactive oxygen species generation. This contributes to the pathogenesis of type 1 diabetes [11]. Similarly, the thyroid gland operates within an iodine-rich oxidative milieu required for thyroid hormone synthesis [12]. In addition, fluctuations in adrenal steroid hormones may affect Treg epigenome and alter *FOXP3* transcriptional stability [13]. These features suggest that endocrine glands represent metabolically demanding and inflammation-prone environments. These factors challenge *FOXP3* epigenetic stability and promote dysregulation of Treg-mediated immune tolerance.

Studying the genetic and epigenetic alterations of *FOXP3* in autoimmune endocrine diseases is essential for several reasons. First, it provides mechanistic insight into disease pathogenesis, identifying mutations and epigenetic changes that compromise Treg-mediated self-tolerance and trigger tissue-specific autoimmunity. Second, it enables the identification of molecular biomarkers for disease susceptibility, progression, and therapeutic response, which is particularly valuable in endocrine autoimmune disorders that often develop silently before clinical onset. Third, understanding *FOXP3* regulation offers a foundation for the development of targeted therapies aimed at restoring immune balance. This will provide new insights into disease pathogenesis and identify potential therapeutic targets aimed at restoring immune tolerance and preventing progression of endocrine autoimmune disorders.

This review discusses the role of *FOXP3* instability in Tregs as a central mechanism driving endocrine autoimmunity. Specifically, it addresses how instability or loss of *FOXP3* expression impair Treg suppressive function in endocrine organs. In addition, molecular, metabolic, and inflammatory signals contributing to Treg plasticity and conversion into pro-inflammatory phenotypes are discussed.

## 2. FOXP3 Gene Structure

**Figure 1:**
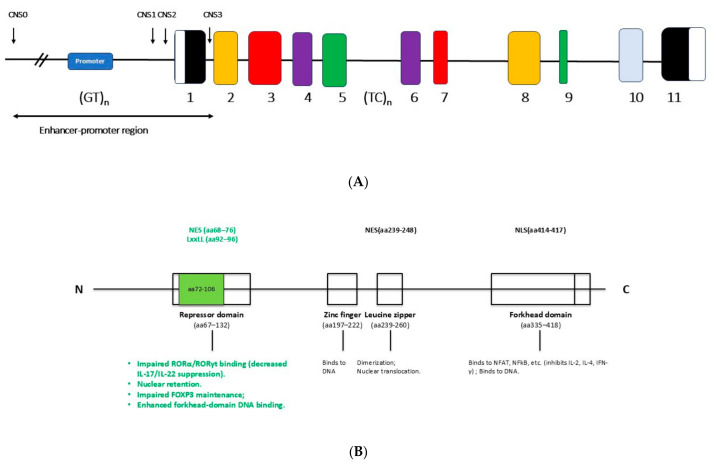
(**A**) Structure of the human *FOXP3* gene. Colored boxes represent coding regions, white boxes indicate non-coding regions, and horizontal lines represent introns. Exon sizes (in base pairs) are shown above each exon. Arrows point at the positions of the (GT)n microsatellite in intron 0, the (TC)n microsatellite in intron 5, and the conserved non-coding sequences (CNS). (**B**) FOXP3 domain structure. Full-length FOXP3 (431 aa, 47 kDa) and FOXP3ΔE2 (396 aa, 43 kDa) are shown. The deleted exon 2 region (aa 72–106) contains motifs involved in interactions with RORα/RORγt and regulation of FOXP3 function. Major functional domains, including the repressor, zinc finger, leucine zipper, and forkhead domains, as well as nuclear localization and export signals, are indicated. NES, nuclear export signal; NLS, nuclear localization signal; LxxLL motif (where *L* represents leucine and *x* represents any amino acid), a multifunctional sequence involved in transcriptional regulation and protein–protein interactions.

The human *FOXP3* gene is located on the X chromosome and consists of one non-coding exon and 11 coding exons (Figure 1). In humans, *FOXP3* consists of 431 amino acids with a molecular weight of 47.25 kDa and contains four functional domains: a repression domain, a zinc finger, a leucine zipper motif, and a forkhead (FKH) domain [14]. *FOXP3* can bind to over 2800 genomic loci, modulating gene expression both positively and negatively at the transcriptional level. Moreover, FOXP3 regulates gene expression post-translationally by modulating protein stability, interactions, and modifications like acetylation or ubiquitination [15,16].

Three conserved non-coding sequences (CNS) contribute to transcriptional regulation of *FOXP3* expression (Figure 1). CNS1 is particularly important for activating *FOXP3* in response to transforming growth factor-beta (TGF-ß) signaling. TGF-ß promotes the recruitment of transcription factors, including SMAD3, and nuclear factor of activated T cells (NFAT), to CNS1 in the *FOXP3* promoter, thereby initiating *FOXP3* transcription [17]. SMAD3 binding to CNS1 is especially critical for the differentiation of induced FOXP3^+^ Treg (iTreg) cells outside the thymus. Mouse models with CNS1 deficiencies exhibit impaired iTreg induction, resulting in a failure to maintain immune tolerance in peripheral tissues such as the intestine [18].

Foxp3 binds TTTG and broader TnG repeats (*n* = 2–5) near accessible chromatin. Murine Foxp3 forms diverse supramolecular assemblies depending on DNA sequence, bridging 2–4 DNA duplexes into ultra stable structures. Nucleosomes promote this assembly by bending DNA, enabling Foxp3 to connect distant loci. These findings reveal Foxp3’s structural flexibility in recognizing variable microsatellites and stabilizing chromatin architecture [19].

## 3. Posttranscriptional Modifications of FOXP3

**Figure 2:**
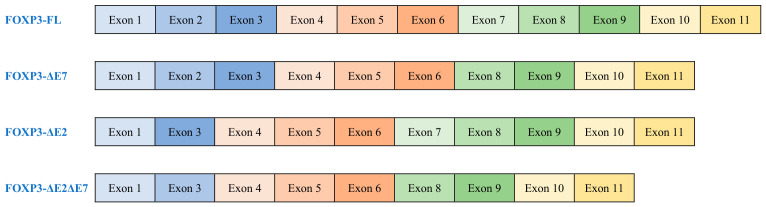
Alternatively spliced isoforms of FOXP3.

Posttranscriptional modifications of FOXP3 involve changes that occur after the *FOXP3* mRNA is produced, affecting its stability, translation, and ultimately the function of regulatory T cells. Alternative splicing generates different FOXP3 isoforms, such as FOXP3-FL, FOXP3-ΔE2, and FOXP3-ΔE7, each with distinct regulatory roles. The full-length FOXP3 (FOXP3-FL) isoform contains all coding exons while the alternatively spliced FOXP3-ΔE2 isoform lacks exon 2 (Figure 2). These 2 forms constitute the two major forms of FOXP3. Two additional minor isoforms have also been described: one lacking exon 7 (FOXP3-ΔE7) and another missing both exons 2 and 7 (FOXP3-ΔE2ΔE7) [20].

The FOXP3 protein interacts with multiple transcriptional regulators and binds to DNA at several sites. Specifically, exon 2 mediates interactions with RORγt, RORα, and hnRNPF, while exon 7 is known to interact with FOXP1, FOXP3 itself, and DNA. The alternatively spliced isoforms exhibit differences in subcellular localization and transcriptional regulator binding domains. Therefore, they have potential functional distinctions that may affect disease pathogenesis [20].

Normally, FOXP3-FL and FOXP3-ΔE2 are expressed at equal levels while in severe systemic autoimmune disorders like immune dysregulation, polyendocrinopathy, enteropathy, X-linked (IPEX) syndrome exclusive expression of FOXP3-ΔE2 was reported [20]. FOXP3-ΔE2 lacks sequences mediating interactions with transcription factors like RORα and RORγt that are involved in helper T 17 (Th17) cell differentiation [21]. Subsequently, FOXP3-ΔE2 is unable to inhibit Th17 differentiation. This leads to an imbalance in Treg/Th17 homeostasis, which is implicated in autoimmune pathology [22].

Interestingly, an alternative *FOXP3* promoter was described recently. This promoter is active only in Tregs and produces a non-coding isoform called long non-coding FOXP3 transcript (lncFOXP3). Its transcription is linked to reduced *FOXP3* mRNA and protein levels. Therefore, this promoter may modulate canonical *FOXP3* expression and fine-tune Treg function [23].

In addition to alternative splicing, FOXP3 undergoes multiple post-translational modifications that regulate its stability and function. Acetylation protects it from degradation and enhances transcriptional activity, while phosphorylation by kinases such as CDK2 or PIM1 can reduce stability and suppressive capacity. Ubiquitination by E3 ligases like STUB1 promotes degradation, whereas methylation by PRMT1 strengthens DNA binding. Together, these modifications dynamically control FOXP3 levels, activity, and Treg-mediated immune tolerance [24]. Chemical modifications such as N6-methyladenosine (m6A) can alter mRNA stability and translation efficiency [25]. Additionally, microRNAs, including miR-31 and miR-155, can bind *FOXP3* mRNA to modulate translation or promote degradation [26].

A clear example of FOXP3 post-translational regulation in Treg stability is seen in Hashimoto’s thyroiditis. Reduced FOXP3 expression and impaired Treg function are linked to abnormal SIRT1-mediated deacetylation, which decreases FOXP3 acetylation, destabilizes the protein, and diminishes suppressive activity. Pharmacologic inhibition of SIRT1 with Ex-527 (selisistat) restores FOXP3 acetylation and increases functional Treg frequency [27].

## 4. The Role of FOXP3 in Maintaining Immune Tolerance and Preventing Endocrine Autoimmunity

Genetic proof in humans comes from IPEX syndrome, a fulminant early-onset autoimmune disorder caused by *FOXP3* mutations. Patients with *FOXP3* loss-of-function develop multi-organ autoimmunity that commonly includes endocrine disease (type 1 diabetes and autoimmune thyroid disease among manifestations), directly linking FOXP3 deficiency to endocrine autoimmunity in humans. IPEX is a life-threatening disorder. Genetic evidence demonstrates that various mutations in the human *FOXP3* gene; the ortholog of the gene mutated in scurfy mice, are responsible for IPEX syndrome. Recent linkage analyses have localized the IPEX-associated gene to a 17–20 cM interval on Xp11.23–Xq13.3 therefore it is a sex-linked disease affecting males [28,29].

Multiple original studies using mouse models of autoimmune diabetes (type 1 diabetes) and human samples implicate Foxp3^+^ Tregs in protection from islet autoimmunity. In non-obese diabetic and other models, loss or functional impairment of Foxp3^+^ Tregs accelerates β-cell autoimmunity; conversely, approaches that expand antigen-specific Foxp3^+^ cells can prevent or slow disease in experimental settings [29,30].

## 5. Treg Cell Stability vs. Plasticity in Endocrine Microenvironment

Treg cell stability refers to the ability of Treg cells to maintain FOXP3 protein expression even under inflammatory stress. This ability prevents them from converting into pro-inflammatory effector cells. In other words, stability implies staying as FOXP3^+^ Tregs and avoiding conversion into FOXP3^−^ ex-Tregs. This stability is controlled by metabolic pathways, such as glycolysis, fatty acid β-oxidation, and amino acid catabolism, epigenetic, and transcriptional regulation [31].

### 5.1. Inflammatory and Metabolic Stressors of Treg Stability in Type 1 Diabetes

Endocrine tissues create microenvironments that challenge Treg stability by imposing metabolic stress, inflammatory cytokines, and fluctuating hormonal signals. In type 1 diabetes (T1D), the pancreatic islet microenvironment is characterized by inflammatory cytokines and reduced availability of interleukin-2 (IL-2), conditions that impair Treg stability and function. Adequate IL-2 signaling is essential for maintaining *FOXP3* gene expression and the survival of Tregs through activation of the STAT5 pathway. In T1D, defects in the IL-2 pathway—including decreased IL-2 production by conventional T cells and reduced expression or signaling of CD25 (IL-2Rα), the high-affinity IL-2 receptor subunit on Tregs—limit the ability of Tregs to efficiently capture IL-2. This impaired IL-2 signaling compromises Treg survival and destabilizes *FOXP3* expression [32,33]. This weakens Tregs’ suppressive capacity and contributing to the breakdown of immune tolerance driving autoimmune destruction of pancreatic β-cells [34].

Pro-inflammatory mediators such as IFN-γ and IL-6 further promote Treg dysfunction and lineage instability within inflamed islets [35,36]. Because Tregs depend heavily on mitochondrial oxidative phosphorylation rather than glycolysis for suppressive activity, mitochondrial dysfunction or metabolic stress can impair their regulatory function [37]. Disruption of mitochondrial homeostasis activates the integrated stress response, including ATF4-dependent transcriptional programs. These programs drive metabolic reprogramming and epigenetic remodeling associated with reduced *FOXP3* stability and diminished suppressive capacity [38].

### 5.2. Genetic, Immunological, and Metabolic Determinants of Treg Instability in Autoimmune Thyroid Disease

Changes in thyroid hormone receptor-α (TRα) signaling in T cells alters Treg activity and cytokine responses [39]. In autoimmune thyroid diseases such as Graves’ disease, elevated thyroid hormone levels have been shown to impair Treg suppressive activity and reduce expression of inhibitory receptors such as PD-1 [40]. Clinical studies have produced inconsistent findings in Graves’ disease, some investigations reported no significant change in Treg numbers, whereas others observed either decreased or increased frequencies of Tregs [41].

A meta-analysis demonstrated that patients newly diagnosed with Hashimoto’s thyroiditis and Graves’ disease have significantly elevated levels of Th17 cells in peripheral blood and lower levels of Treg and *FOXP3* mRNA compared to the healthy population [42]. Th17 cells are pro-inflammatory lymphocytes producing IL-17A/F and IL-21, promoting tissue inflammation and autoimmunity. Their expansion in AITD is closely associated with dysfunction of *FOXP3*-expressing Tregs, which maintain immune tolerance. Reduced number or impaired function of Tregs disrupts the Treg–Th17 balance, favoring pro-inflammatory Th17 responses. In thyroid tissue from Hashimoto’s thyroiditis patients, IL-17 expression has been detected in both follicular cells and inflammatory infiltrates and is associated with follicular damage and loss of structural integrity [43]. Therefore, the observed increase in Th17 cells in AITD likely reflects a breakdown of *FOXP3*-dependent Treg regulation, contributing to autoimmune inflammation and thyroid tissue damage [43]. A recent study showed that the single nucleotide polymorphism rs3761548 and rs3761549 in the promoter region of *FOXP3* showed a higher frequency in the comparison of genotype “CT” only in Hashimoto’s thyroiditis patients than in the healthy population [42].Therefore, the imbalance in Th17/Treg ratio may result in autoimmune thyroid disease.

A recent study reported that tryptophan levels are reduced in Hashimoto’s thyroiditis compared with controls [44]. In a C57BL/6 mouse Hashimoto’s thyroiditis model, tryptophan supplementation attenuated thyroid tissue damage, reduced inflammatory cytokine production, and restored immune homeostasis, whereas pharmacological inhibition of tryptophan metabolism exacerbated thyroid injury and inflammation. Furthermore, tryptophan influenced the balance of T-cell subsets and reshaped the immune microenvironment, highlighting its immunomodulatory properties. Mechanistic analyses indicated that these protective effects were associated with modulation of immune-related gene expression and regulation of the PI3K–Akt signaling pathway, suggesting that dysregulated tryptophan metabolism may contribute to Hashimoto’s thyroiditis progression through interconnected effects on immune tolerance, inflammatory responses, and intracellular signaling pathways [44].

Taken together, these findings support a unified model in which impaired FOXP3 expression or function disrupts Treg-mediated immune tolerance in autoimmune thyroid disease. Reduced Treg suppressive capacity permits the expansion of pro-inflammatory Th17 cells and increased production of cytokines such as IL-17, which contribute to thyroid inflammation and tissue injury. Genetic factors, including *FOXP3* promoter polymorphisms, may further compromise Treg stability and function, thereby predisposing susceptible individuals to disease development. In addition, metabolic alterations, such as reduced tryptophan availability, may influence Treg differentiation and FOXP3 expression, further shifting the immune balance toward Th17-mediated inflammation. Thus, genetic susceptibility, immune dysregulation, and metabolic disturbances appear to converge on a common pathway characterized by impaired FOXP3-dependent Treg function and loss of immune tolerance, ultimately promoting the initiation and progression of autoimmune thyroid disease.

### 5.3. Endocrine Hormones as Modulators of Treg Stability and Plasticity

Treg cell stability and plasticity are dynamically regulated by the endocrine microenvironment, where hormonal fluctuations critically shape immune tolerance and susceptibility to autoimmunity. Gonadal sex hormones act as a key link between endocrine signaling and immune regulation by modulating Treg differentiation and stability. During pregnancy, high estrogen (E2) acting through estrogen receptor-α (ERα)-dependent signaling pathways that enhance *FOXP3* transcription and Treg differentiation, contributing to maternal immune tolerance [45]. After delivery, the marked decline in estrogen and progesterone is associated with a reversal of pregnancy-induced immune tolerance leading to the postpartum exacerbation or relapse of autoimmune diseases, including autoimmune thyroid disorders such as Graves’ disease and postpartum thyroiditis [46].

In contrast, not all autoimmune endocrine diseases show the same degree of Treg instability, as illustrated in other organ-specific conditions. Primary adrenal insufficiency is mainly caused by autoimmune destruction of the adrenal cortex. Although Treg cells are often altered in autoimmune diseases, patients with primary adrenal insufficiency showed similar Treg frequency and function as controls, suggesting Tregs likely do not play a major role in primary adrenal insufficiency development [47].

## 6. Genetic Determinants of FOXP3 Dysfunction

Genetic alterations affecting *FOXP3* expression and isoform balance represent a key mechanism underlying Treg dysfunction in autoimmune endocrine diseases. In conditions such as Hashimoto’s thyroiditis, perturbations in *FOXP3* isoform expression patterns were reported. For instance, an increased FOXP3-ΔE2 isoform proportion correlated with defective Treg suppression, contributing to loss of self-tolerance and enhanced autoreactive T cell activity [14,48].

Sex-linked genetic regulation further contributes to *FOXP3*-mediated immune control. Females are more susceptible to autoimmune diseases than males [49]. The quantity and functional activity of Treg cells are regulated by X-linked *FOXP3* and hormonal fluctuations [50]. Because one of the two X chromosomes is randomly inactivated, it was assumed that if a mutation occurs in the *FOXP3* gene on one X chromosome, the resulting Treg cells may be functionally defective. However, it was found that female carriers of an IPEX-causing mutation in the *FOXP3* gene are typically healthy because their peripheral blood lymphocytes show random X-chromosome inactivation, with no preferential inactivation of the chromosome with the mutated allele [51]. Therefore, expression of the wild-type *FOXP3* gene from the other X chromosome allows the generation of normal Treg cells, offering protection against the development of autoimmune disease.

### 6.1. IPEX Syndrome and Early-Onset Insulin-Requiring Diabetes

IPEX syndrome has been recognized as the prototype of autoimmune monogenic diseases. IPEX classic form presents within the first year of life with severe atopic dermatitis, insulin-dependent type 1 diabetes, and autoimmune enteropathy. Affected individuals often exhibit excessive allergic and immune activation characterized by circulating autoantibodies against target organs, elevated lymphocyte and eosinophil counts, and markedly increased serum immunoglobulin E [52]. Since polyglandular autoimmune syndromes (except IPEX-like cases) are not driven by *FOXP3* defects [53], it falls outside the scope of this review.

Multiple types of pathogenic mutations in the *FOXP3* gene involving both coding and non-coding regions have been identified in IPEX and related immune dysregulation syndromes. Among 102 previously negative male patients, 8 (7.8%) were found to carry previously undetected pathogenic *FOXP3* variants, demonstrating that next-generation sequencing can uncover clinically significant *FOXP3* mutations including splice site, deletion, and missense variants [54]. Importantly, missense mutations like R386H and R379Q demonstrate marked phenotypic variability among affected individuals. For instance, the R379Q mutation may manifest with classic IPEX symptoms such as severe, early-onset diarrhea, life-threatening dermatitis, hemolytic anemia, and recurrent infections, but in others the presentation may be milder or feature different autoimmune endocrinopathies, highlighting genotype–phenotype heterogeneity [55,56]. Even siblings sharing the same mutation both with the c.-23G>A splice site mutation may have different presentation ages and autoimmunity spectrums. This underlines the influence of modifying genetic and environmental factors as well as possible X-inactivation effects in carrier females [51]. The clinical heterogeneity that ranges from fatal neonatal presentations to later-onset, moderate multisystem autoimmunity is supported by multiple patient case reports and referenced molecular analyses [57,58]. A detailed summary of the reported *FOXP3* variants, their genomic positions, and associated phenotypes is provided in Supplementary Table S1.

To explore whether *FOXP3* mutations are responsible for early-onset insulin-dependent diabetes, particularly in the absence of typical IPEX features, the 11 coding exons and the polyadenylation region of the *FOXP3* gene were sequenced in 26 male infants diagnosed with diabetes before 6 months of age. Among these patients, 10 exhibited additional immune-related disorders, whereas 16 presented with isolated diabetes. Hemizygous *FOXP3* mutations were identified in 6 of the 10 patients with immune manifestations but in none of the 16 with isolated diabetes. Three patients carrying two novel missense variants (R337Q and P339A) and one previously reported frameshift mutation (L76QfsX53) developed classic IPEX syndrome and died within 13 months of age. Another missense mutation (V408M) was detected in three patients from two unrelated families, who showed a milder phenotype characterized by hypothyroidism and autoimmune enteropathy in two cases, and nephrotic syndrome in one case, with survival extending to 12–15 years [59].

In a study investigating the association of *FOXP3* with type 1 diabetes, researchers identified two microsatellite regions in *FOXP3*; intron 0 and intron 5. Comparison of allele distributions in 26 patients for the intron 0 polymorphism between patients and controls revealed a significant difference (*p* = 0.034), indicating a possible association between the *FOXP3* (also known as *Scurfin*) and type 1 diabetes. Specifically, the (GT)_15_ allele was more frequent in patients than in controls (43.1% vs. 32.6%, *p* = 0.0027) [60]. Larger case–control analyses failed to confirm these associations, indicating that *FOXP3* does not substantially influence type 1 diabetes susceptibility [61]. To sum up, rare *FOXP3* mutations cause monogenic early-onset diabetes, but common *FOXP3* variants or microsatellites have limited impact on typical type 1 diabetes.

Although polygenic autoimmune diabetes (type 1 diabetes) is the most common form of diabetes in children, monogenic variants causing autoimmune diabetes have also been reported. Because both forms share overlapping clinical features, monogenic autoimmune diabetes is sometimes misdiagnosed as type 1 diabetes. Among these, *FOXP3* mutations are a major cause of early-onset, insulin-requiring diabetes associated with immune dysregulation, but not of isolated diabetes. Loss-of-function *FOXP3* variants disrupt Treg activity. This leads to autoimmune destruction of pancreatic β-cells either as an isolated manifestation or as part of broader syndromes such as IPEX. Hemizygous *FOXP3* mutations—including p.Arg114Trp, p.Arg347His, p.Lys393Met, and the splice-site variant c.1044+5G>A—have been identified in male infants with early-onset diabetes [62]. The splice-site mutation results in aberrant RNA splicing and truncated *FOXP3* transcripts, compromising Treg function and triggering diabetes onset.

Recently, RNA-based analyses have confirmed that even non-coding splice-site variants, such as c.-22-2delA, can disrupt *FOXP3* transcription and lead to early-onset autoimmune diabetes [1]. These findings underscore the critical role of FOXP3 in immune homeostasis and the importance of *FOXP3* sequencing in infants presenting with neonatal or early-onset insulin-dependent diabetes accompanied by immune-related abnormalities. Conversely, several studies have reported no significant differences in *FOXP3* variants or in the frequency of FOXP3^+^ Treg cells between type 1 diabetes patients and controls, indicating that FOXP3 is unlikely to play a major genetic role in typical type 1 diabetes. Moreover, no changes in FOXP3^+^ T-cell frequency were observed in type 1 diabetes, and genotyping of six *FOXP3* single-nucleotide polymorphisms (SNPs) across multiple cohorts revealed no significant associations with the disease [63,64,65].

### 6.2. Autoimmune Thyroid Diseases

Genetic studies on *FOXP3* single nucleotide polymorphisms (SNPs) have identified significant associations with autoimmune thyroid diseases (AITDs), predominantly Graves’ disease and Hashimoto’s thyroiditis, with observed ethnic variability. Several promoter and intronic SNPs, such as rs3761548, rs3761549, rs3761547, showed significant associations with Graves’ disease susceptibility, severity, and response to treatment. However, some studies failed to find such association (Table 1). The AA genotype of rs3761548 was consistently more frequent in Graves’ disease and linked to severe disease or female predisposition [66,67]. Promoter variants −3279 C/A (corresponds to SNP rs3761548) and −2383 C/T (corresponds to SNP rs3761549) have been strongly linked to Graves’ disease, with carriers of the mutant alleles showing reduced *FOXP3* expression and promoter activity [68,69]. Meta-analyses further identified rs3761548 and rs3761549 as risk markers, particularly among Asian populations [70,71], whereas rs3761547 appeared largely irrelevant. Ethnic differences were evident: no significant associations were found in British, Italian, or Japanese cohorts [2,3,72]. Notably, the −3279 AA genotype (SNP rs3761548) was absent in Japanese patients in remission, suggesting it may contribute to persistent disease [3].

In Hashimoto’s thyroiditis, the rs3761548 CC genotype increased susceptibility and correlated with higher anti-thyroid peroxidase antibody (anti-TPO) levels in Iranians [73] while Polish patients had no significant associations [74]. In a study conducted in India, rs3761548 and rs3761549 variants were not directly linked to AITD risk. Haplotype analysis showed that the combination of rs3761548 “C” and rs3761549 “T” was more frequent in Hashimoto’s thyroiditis and Graves’ disease patients, suggesting it may increase disease risk (*p* = 0.03) [75]. Possible gene–gene interactions, between *FOXP3* and *CTLA-4* loci was suggested since the “G” allele of rs231775 (for *CTLA-4*) significantly increased the risk of developing Hashimoto’s thyroiditis (*p* = 0.009) and Graves’ disease (*p* = 0.02). Therefore, it was concluded that *CTLA-4* and *FOXP3* genes may work together in affecting AITD susceptibility [75]. Table 1 provides a summary of studies investigating the role of *FOXP3* polymorphisms in thyroid autoimmunity. Discrepancies among studies investigating thyroid-related SNPs may be attributed to variations in ethnicity, population structure, sample size, diagnostic criteria, age distribution, and the extent of functional validation. Future studies should account for these factors to improve consistency and reproducibility of findings.

**Table 1:** *FOXP3* polymorphism in autoimmune thyroid diseases.

Autoimmune Endocrine Disease	Design/Sample Size	Ethnicity/Region	SNP	Significant Associations	Non-Significant Findings	Key Notes/Observations	Reference
Graves’ disease	Case–control (66 GD patients/37 controls)	Arab/Iraqi	rs3761548	AA genotype more common in GD (78.9% vs. 21.1%; OR = 3.93, *p* = 0.033); dominant model (AC + AA) significant (OR = 3.01, *p* = 0.0096)		AA genotype associated with severe GD; AC/CC genotypes showed better response to carbimazole	[66]
Graves’ disease with ophthalmopathy (GO) or without GO	Case–control (100 GO/74 GD/100 controls)	Turkish	rs3761547 rs3761548 rs3761549	rs3761548 AC and AA genotype and rs3761549 CT genotype more frequent in patients than controls.	No differences for rs3761547 between GD and controls.	There was no statistically significant difference between GD with or without ophthalmopathy concerning the allele and genotype frequencies of all three SNPs.	[76]
Graves’ disease	Two-stage case–control (503 GD patients/890 controls)	Southwest Chinese	rs3761547 rs3761548 rs3761549 rs2280883	For rs3761548, increased frequency of A allele (*p* < 0.001, OR = 1.672), AA genotype (*p* = 0.005, OR = 2.488) and decreased C allele (*p* = 0.031, OR = 0.611) in females.	rs3761549, rs3761547, and rs2280883 showed no associations.	In females, rs3761548 C allele (OR = 0.62) and CC genotype (OR = 0.62) protective against GD.	[67]
Graves’ disease	Meta-analysis (4051 GD patients/4569 controls)	Asians, Caucasians	rs3761547 rs3761548 rs3761549	rs3761548 A vs. C (OR = 1.32, 95% CI 1.05–1.67); rs3761549 TT vs. CC (OR = 1.98, 95% CI 1.49–2.65); TT + TC vs. CC (OR = 1.44, 95% CI 1.11–1.88)	rs3761547 showed no association	rs3761548 significant only in Asians; rs3761549 significant in both Asians and Caucasians	[70]
Graves’ disease	Meta-analysis (3104 GD patients/3599 controls)	Asians, Caucasians	rs3761547 rs3761548 rs3761549	rs3761548 (OR = 1.31, 95% CI 1.04–1.64, *p* = 0.02); rs3761549 (OR = 1.30, 95% CI 1.03–1.64, *p* = 0.03)	rs3761547 not associated (OR = 1.07, *p* = 0.18)	rs3761548 and rs3761549 associated with increased GD risk in Asians; limited data for rs3761547 in Caucasians	[71]
Graves’ disease	Case–control, 135 GD patients and 150 healthy controls	Kashmiri	*FOXP3* promoter SNPs (−3279 C/A, −2383 C/T, −3499 A/G)	−3279 C/A SNP (OR = 3.48; 95% CI: 2.05–5.92; *p* < 0.001); −2383 C/T SNP (OR = 5.62; 95% CI: 2.43–13.00; *p* < 0.001).	No significant association was observed for the −3499 A/G SNP.		[77]
Graves’ disease	Case–control (110 GD patients/110 controls)	Chinese	C-2383T, C-3279A, A-3499G, T+459C	*FOXP3* expression was lower in GD patients (*p* < 0.0001). C-2383T, C-3279A, and T+459C were significantly higher in GD patients compared to healthy controls.		Wild-type alleles associated with higher expression and mutant alleles linked to reduced expression.	[69]
Graves’ disease	Case–control (308 GD patients and 306 healthy controls)	Chinese	−2383, −3279, −3499 in the promoter, IVS9 + 459	The AA/CA genotype of −3279 and CC genotype of IVS9+459 were significantly higher in GD. −3279 A allele more common in females. in patients with higher TSH or lower TSH receptor antibody levels.		Haplotype analysis showed CCA was protective, while CAA and TCA increased GD susceptibility. The −3279 C→A mutation reduced *FOXP3* promoter activity and decreased FOXP3 expression.	[68]
Graves’ disease	Case–control study; 145 GD and 161 controls	Caucasian Polish children and adolescents	rs3761549, rs3761548 rs3761547	In females, rs3761549 G/A more frequent in GD than controls (15% vs. 7%, *p* = 0.033, OR = 2.15, 95% CI: 1.07–4.63). rs3761547 T/C borderline significant in females (13% vs. 7%, *p* = 0.066, OR = 1.99, 95% CI: 0.96–4.48).	No significant differences in males or for rs3761548 SNP.	rs3761549 G/A polymorphism contributes to GD susceptibility in females.	[74]
Graves’ disease	Case–control; 65 intractable GD, 44 GD in remission, and 71 healthy controls	Japanese population	−3279 C/A −3279 AA −2383 CC	−3279 C/A genotype more frequent in GD remission than in intractable GD; −3279 AA genotype absent in GD remission		Suggests −3279 C/A and −2383 C/T polymorphisms influence disease severity and remission status in AITD	[3]
Graves’ disease	Case–control study; 333 GD and 117 controls	Caucasian patients of Italian origin	rs6609857 rs2294021 rs2280883 rs2232365 rs3761549		No significant association with the disease	Ethnic variation in the association of different polymorphisms with GD	[72]
Graves’ disease	Case–control (633 GD patients/528 controls)	UK	rs3761549 (C/T) rs2232365 (G/A) rs2280883 (T/C) rs2294021 (C/T) rs6609857 (C/T)		No significant associations for any of the studied SNPs	No evidence of *FOXP3* variants influencing GD susceptibility	[2]
Hashimoto’s thyroiditis (HT)	Case–control; 129 HT patients and 127 healthy controls	Iranian population	rs3761548	rs3761548 CC genotype associated with increased HT (OR = 2.1, 95% CI: 1.2–3.6, *p* < 0.008); higher anti-TPO antibody levels in CC genotype carriers (*p* < 0.004)	rs3761549 showed no significant allelic association.	C allele and CC genotype increase HT risk; AC genotype linked to lower anti-TPO levels	[73]
Autoimmune thyroid diseases (HT and GD)	Case–control; 355 AITD patients (275 HT, 80 GD) and 285 controls	Indian population	rs231775 rs3761548 rs3761549	rs231775 G allele associated with HT (*p* = 0.009) and GD (*p* = 0.02); haplotype rs3761548 and C–rs3761549 T increased disease risk (*p* = 0.03)	No allelic association for rs3761548 and rs3761549 individually.	Epistatic interaction between *CTLA-4* and *FOXP3* genes influences AITD susceptibility	[75]
Hashimoto’s thyroiditis (HT)	Case–control study; 87 HT and 161 controls	Caucasian Polish children and adolescents	rs3761549, rs3761548 rs3761547		No significant differences		[74]
Hashimoto’s disease	Case–control; 38 severe HT, 40 mild HT and 71 healthy controls	Japanese population	−3279 C/A −3279 AA −2383 CC	−2383CC genotype more frequent in severe than mild HT		Suggests −3279C/A and −2383C/T polymorphisms influence disease severity and remission status in AITD	[3]
Autoimmune thyroid diseases (AITD: GD and HT)	Case–control; 269 Caucasian AITD patients (52 males, 217 females) and 357 Caucasian controls; 377 Japanese female AITD patients and 179 Japanese controls	U.S. Caucasian and Japanese populations		In Caucasians: (TC)n microsatellite associated with AITD in males (*p* = 0.011); DXS573 associated with AITD in females (*p* = 0.00023).	No association found in Japanese cohort.	*FOXP3* locus polymorphisms associated with AITD susceptibility in Caucasians but not in Japanese; suggests ethnic variability in genetic risk.	[78]

Abbreviations: AITD: Autoimmune thyroid diseases; CI: confidence interval; GD: Graves’ disease; HT: Hashimoto’s thyroiditis; OR: odds ratio; p: probability, anti-TPO: anti-thyroid peroxidase antibody.

### 6.3. Other Autoimmune Endocrine Diseases

In a case–control study in the UK, no significant associations for any *FOXP3* single nucleotide polymorphisms and Addison’s disease were found [2]. Autoimmune hypothalamitis and hypopituitarism have been reported [4,79]. However, the role of *FOXP3* gene polymorphisms in their pathophysiology has not yet been explored.

## 7. Epigenetic Regulation of FOXP3 Gene Expression

Epigenetics refers to the heritable changes in gene expression that happen without changing the DNA sequence [80]. These modifications are mediated through multiple molecular mechanisms, including DNA methylation, histone modifications, and interactions with non-coding RNAs. Collectively, these epigenetic mechanisms allow cells to respond to environmental cues, preserve cellular identity, and control complex developmental processes. Epigenetic modifications can play key roles in disease pathogenesis, including autoimmune disorders.

### 7.1. DNA Methylation of FOXP3 Promoter

During thymic development, the methylation status of CNS2 is determined. CNS2 is located between the promoter and enhancer regions and is a key regulatory DNA element [81]. T cell progenitors exhibiting hypomethylation of the *FOXP3* CNS2 region and stable *FOXP3* expression differentiate into Tregs, while those with methylated *FOXP3* CNS2 give rise to effector T cells (Teffs) [82]. Deleting Sin3a (Switch-Independent 3A), a transcriptional corepressor protein, during development leads to rapid, fatal autoimmunity in mice. This deletion significantly reduces Treg numbers, and the remaining Tregs show severely impaired suppressive function. Mechanistically, Sin3a deficiency lowers *FOXP3* transcription and completely blocks CNS2 CpG demethylation, a key epigenetic mark for Treg stability. Furthermore, *FOXP3* protein becomes unstable. These cells, called ex-Tregs, no longer function as regulatory cells and can adopt pro-inflammatory characteristics, contributing to autoimmune pathology [83].

DNA methylation, the addition of a methyl group to the 5′ position of cytosine residues, usually leads to gene silencing. Latent autoimmune diabetes in adults (LADA) represents an intermediate form of diabetes, sharing features of both type 1 (autoimmunity) and type 2 (adult onset, slow progression). In LADA patients *FOXP3* expression was reduced, and its promoter region was hypermethylated. In this disease, significantly higher global DNA methylation and increased DNA methyltransferase 3B (DNMT3B) expression was described [84]. A study that included 20 LADA patients and 20 healthy controls found that DNA methyltransferase 1 *DNMT1*, and *DNMT3b* mRNAs were significantly upregulated in Treg cells from LADA patients compared with controls. The *FOXP3* promoter was significantly hypermethylated in LADA than controls and *FOXP3* mRNA was significantly downregulated [85].

TSDR (Treg-Specific Demethylated Region) is a key epigenetic marker within the CNS2 of the *FOXP3* gene locus that determines the stability and lineage identity of Tregs. Normally, demethylation of the TSDR is associated with stable *FOXP3* expression and functional Treg development. Paradoxically, higher TSDR demethylation was reported in IPEX [86]. In fact, this results from an expansion of *FOXP3*-mutated, nonfunctional Treg-like cells. Similar findings were obtained in a cohort of 65 genetically confirmed IPEX patients where the percentage of demethylated *FOXP3* TSDR/CD4 cells effectively distinguished IPEX patients from controls (ROC-AUC = 0.81) [87].

Interestingly, reduction in *FOXP3* gene methylation levels among Hashimoto’s thyroiditis patients was possible using dietary interventions. This was achieved through following a diet excluding casein and gluten for 3 months. Controls who maintained normal dietary guidelines showed no significant alterations in methylation levels [88]. Therefore, nutritional interventions could potentially be a strategy for mitigating autoimmunity through epigenetic mechanisms.

### 7.2. Histone Modifications Influencing FOXP3 Transcription

Histone modifications such as acetylation, methylation, phosphorylation, and ubiquitination modulate chromatin structure and accessibility, thereby influencing transcriptional activity. Active histone marks enrich at the *FOXP3* promoter and enhancer elements in Tregs [89].

Patients with Graves’ disease and Hashimoto’s disease showed significantly reduced histone H3 acetylation compared to controls [90]. In LADA, histone H3 acetylation at K9 and K14 of the *FOXP3* promoter was significantly lower than controls resulting in marked *FOXP3* mRNA downregulation. HDAC3 and HDAC5 histone deacetylases’ mRNAs were significantly upregulated in Treg cells of LADA patients [85].

### 7.3. Non-Coding RNAs and FOXP3 Regulation

Non-coding RNAs, including microRNAs (miRNAs) and long non-coding RNAs (lncRNAs), regulate gene expression post-transcriptionally by affecting mRNA stability or translation in Treg cells. For example, miR-31 directly targets the 3′-UTR of *FOXP3* mRNA, suppressing its translation and impairing Treg differentiation [91]. miR-31 drives thyroid cancer progression [92]. However, the role of miR-31 in the pathogenesis of different endocrine autoimmune diseases has not been fully investigated.

In mouse Treg cells, *Foxp3* directly promotes the expression of miR-155, while miR-155, in turn, regulates key aspects of Treg function. Specifically, miR-155 suppresses the translation of SOCS1, a negative regulator of cytokine signaling, thereby enhancing IL-2 sensitivity and giving Tregs a competitive advantage in proliferation and survival [93]. However, this regulatory mechanism appears to differ in the HOZOT cell line, which has low and stable miR-155 expression and retains normal IL-2 responsiveness. Bioinformatic and functional analyses revealed that miR-155 targets *FOXO3a* (full-length *FOXO3*), a negative regulator of Akt signaling, which influences Treg survival, proliferation, and function [26].

In non-obese diabetic (NOD) mice, the miR-142-3p/Tet2/*Foxp3* axis regulates Treg lineage stability by controlling epigenetic demethylation of the *Foxp3* locus. High miR-142-3p reduces Tet2, causing *Foxp3* silencing. Blocking miR-142-3p restores Tet2, boosts stable Tregs, and helps prevent autoimmune diabetes [94].

In Graves’ disease, reduced miR-23a-3p leads to increased SIRT1 expression, which decreases *FOXP3* acetylation and impairs Treg function. Restoring miR-23a-3p suppresses SIRT1, enhances *FOXP3* expression and acetylation, and improves Treg suppressive activity, highlighting a miR-23a-3p–SIRT1–FOXP3 regulatory axis in Treg dysfunction [95]. Investigating how miRNAs regulate *FOXP3* in autoimmune endocrine diseases remains an underexplored area that warrants further research.

## 8. Genetic and Epigenetic Determinants of FOXP3 Instability in Treg Cells

Genetic and epigenetic modifiers critically regulate *FOXP3* locus accessibility and Treg lineage stability. The chromatin organizer SATB1 orchestrates higher-order chromatin architecture and primes Treg-specific super-enhancers at the *FOXP3* locus during thymic Treg development. SATB1 binds at regulatory regions such as CNS0, facilitating enhancer activation and chromatin looping that precedes *FOXP3* transcription. Unlike conventional transcription factors that preferentially bind accessible chromatin, SATB1 can associate with relatively closed chromatin during the double-positive thymocyte stage. This early binding event establishes the chromatin architecture required for Treg-cell development. Conditional loss of SATB1 from the double-positive stage disrupts activation of Treg-specific super-enhancers, Treg differentiation and causes severe systemic autoimmunity. Mechanistically, SATB1 cooperates with the enhancer-priming histone methyltransferase MLL4 at a conserved regulatory element upstream of the *FOXP3* locus, designated CNS0. This early enhancer priming subsequently facilitates sequential activation of additional regulatory elements, including CNS3 and CNS2, followed by activation of the *FOXP3* promoter, thereby establishing the transcriptional program required for stable Treg lineage commitment [96,97].

In mature Tregs, stable *FOXP3* expression depends on DNA demethylation of the CNS2 region (TSDR). This permits binding of transcriptional complexes containing RUNX1 and its cofactor CBFβ to form a feed-forward circuit that maintains *FOXP3* transcription and lineage stability [98]. Histone acetyltransferases such as p300 and CBP promote *FOXP3* expression by acetylating enhancer regions, particularly through the histone mark H3K27ac. They also interact cooperatively with key Treg transcription factors, strengthening the transcriptional programs required to maintain regulatory T cell identity [99]. Enhancer of zeste homolog 2 (EZH2), a histone H3 lysine 27 (H3K27) methyltransferase, catalyzes H3K27me3 at non-Treg loci (e.g., *Ifng*, *Il17a*), synergizing with *FOXP3* to block erosion and enforce Treg stability. EZH2 is crucial for Treg differentiation and T effector cell expansion [100]. Enhancing EZH2 activity increases H3K27me3-mediated repression, which strengthens Treg suppressive function by promoting early effector Treg differentiation [101]. In addition, Blimp-1 helps maintain Treg identity by establishing epigenetic barriers at inflammatory gene loci. Loss of Blimp-1 disrupts these barriers and promotes Foxp3-to-RORγt reprogramming, leading to the emergence of Th17-like inflammatory cells [102].

Unlike mouse *Foxp3* that produces single gene product, human *FOXP3* produces two isoforms by alternative splicing: the full-length form and a shorter form lacking exon 2 (*FOXP3* ΔE2). Mice with *Foxp3* exon 2 deletion developed exaggerated T follicular helper and germinal center B cell responses, leading to unstable Treg identity and spontaneous autoimmunity [103]. Also, IPEX patients expressing only the shorter isoform fail to maintain self-tolerance and develop autoimmune responses [103]. These findings suggest that *Foxp3* ΔE2 is a critical stability domain.

The RNA-binding protein HuR (Elavl1), a key post-transcriptional regulator, plays a critical role in T cell activation and function by stabilizing target mRNAs. HuR directly stabilizes *Foxp3* mRNA in Treg nuclei. HuR ablation in Foxp3YFP-Cre HuRfl/fl mice decreased Foxp3 protein levels by over 70%. This skews Tregs toward IFN-γ-producing ex-Tregs and elicits scurfy-like autoimmunity with dermatitis, colitis, and lymphadenopathy. This demonstrates that post-transcriptional regulation is a key genetic checkpoint [104].

## 9. Reversibility vs. Irreversibility of FOXP3 Loss

*FOXP3* instability is increasingly recognized as a dynamic and context-dependent process in which Tregs may undergo reversible functional plasticity before progressing to irreversible dysfunction or exhaustion. Experimental models using inducible AID-degron *FOXP3* knockouts have demonstrated that inflammatory signals can transiently destabilize *FOXP3* expression. For example, acute TNF-α exposure induces reversible epigenetic remodeling and demethylation of pro-inflammatory loci. On the other hand, restoration of IL-2 signaling partially rescues *FOXP3* expression and Treg suppressive function. In contrast, complete genetic deletion of *FOXP3* results in irreversible conversion of Tregs into pathogenic Th1/Th17-like effector cells [105,106].

*FOXP3* interacts dynamically with chromatin, exhibiting relatively short residence times that allow flexible transcriptional regulation and functional plasticity. Persistent inflammatory signaling can destabilize this network and promote lineage erosion, particularly when transcriptional regulators such as SATB1 are disrupted [97]. Epigenetic interventions aimed at restoring Treg lineage stability have demonstrated that activation of TET-dependent DNA demethylation pathways can partially restore demethylation of the conserved non-coding sequence 2 (CNS2) region of the *FOXP3* locus in dysfunctional human Tregs. This leads to a measurable recovery of suppressive function, although the degree of reversibility appears to depend on the proliferative and inflammatory history of the cells [107].

Once established, pro-inflammatory transcriptional circuits—including T-bet/IFN-γ-driven loops—can stabilize pathogenic phenotypes and limit reversibility, although early epigenetic dysfunction in autoimmune settings such as systemic lupus erythematosus has been partially reversed using demethylating agents such as 5-aza-2′-deoxycytidine [108]. Together, these findings indicate that *FOXP3* loss exists along a spectrum from reversible plasticity to irreversible lineage instability, with important implications for therapeutic strategies aimed at restoring immune tolerance.

## 10. Integrated Genetic–Epigenetic Model of FOXP3 Erosion

*FOXP3* expression and function are regulated by a complex interplay of genetic, epigenetic, and metabolic mechanisms. A cohort identified 44 distinct *FOXP3* variants among 88 IPEX patients, underscoring the phenotypic heterogeneity associated with *FOXP3* mutations [109]. Clinically, *FOXP3* mutations cause IPEX syndrome, which can show a wide range of symptoms or disease severity [110]. Even siblings with the identical mutation may show strikingly different symptoms [55].

Modulation of metabolic pathways can affect *FOXP3* transcription and splicing [14]. For example, glycolysis controls the induction of human Treg cells by modulating the expression of FOXP3-ΔE2 splicing variants [111]. Another example is that butyrate induces Treg differentiation primarily via T cell intrinsic epigenetic regulation of the *FOXP3* gene. This involves the inhibition of HDACs, leading to enhanced histone acetylation at the *FOXP3* locus and stabilizing *FOXP3* expression in inducible Tregs [112]. Therefore, targeting metabolic pathways and *FOXP3* splicing variants in Treg cells could offer novel therapeutic strategies for autoimmune diseases.

## 11. FOXP3 as a Biomarker for Autoimmune Endocrine Disease Susceptibility and Progression

IPEX is difficult to diagnose due to its variable symptoms and overlap with other autoimmune diseases. Identifying *FOXP3* mutations can confirm a diagnosis of IPEX syndrome (Table 1) [53]. However, there is a challenge of interpreting novel *FOXP3* mutations. It has been suggested that an elevated percentage of demethylated *FOXP3* TSDR/CD4 serves as a biomarker for diagnosing IPEX and monitoring disease progression [87].

Measuring the balance of *FOXP3* isoforms in blood or affected tissues can help track disease activity and monitor how well treatments are working in autoimmune endocrine disorders [14,48]. An increased FOXP3-ΔE2 isoform proportion in Hashimoto’s thyroiditis and Graves’ disease was reported. This elevation correlated with defective Treg suppression, contributing to loss of self-tolerance and enhanced autoreactive T cell activity [48].

## 12. Therapeutic Strategies Targeting FOXP3

**Figure 3:**
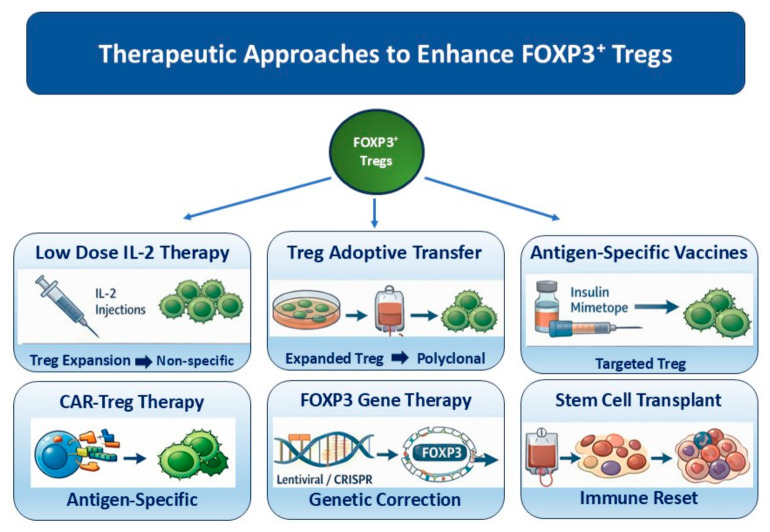
Therapeutic approaches to enhance FOXP3^+^ Treg.

An imbalance in Treg activity or number can have important immunological consequences. Excessive Treg function or expansion may promote immunodeficiency, persistent infections, and tumor progression, whereas reduced Treg activity or numbers can lead to autoimmunity, immunopathology, and impaired pathogen-specific immune responses [113]. To address these issues, cell-based therapies have gained attention (Figure 3). For example, engineered CAR-Tregs have shown remarkable efficacy in disease models, and strategies such as low-dose IL-2 therapy or Treg adoptive cell transfer have been applied in patients with type 1 diabetes [114].

In type 1 diabetes, the immune system mistakenly attacks pancreatic β-cells. Normally, FOXP3^+^ Tregs control such autoreactive responses. Individuals at-risk often have insufficient or unstable insulin-specific Tregs. Researchers developed a strategy using low-dose insulin mimetope vaccines to selectively induce insulin-specific FOXP3^+^ Tregs in mice. These induced Tregs are stable and can suppress harmful effector T cells. By generating antigen-specific Tregs, this approach restores immune tolerance precisely at the target antigen, offering a safe and targeted method to prevent islet autoimmunity and the onset of type 1 diabetes in children at risk [115]. This motivates researchers to expand functional FOXP3^+^ Tregs in humans.

Currently, the main therapeutic option for patients with IPEX syndrome is allogeneic hematopoietic stem cell transplantation. For those who are not candidates for hematopoietic stem cell transplantation, immunosuppressive therapy combined with long-term supportive care is considered. Hematopoietic stem cell transplantation is performed using cells from HLA-identical siblings or other HLA-matched donors. The stem cell source may include cord blood, peripheral blood, or bone marrow [116].

Insulin-specific chimeric antigen receptors (CAR)-Tregs were generated by transducing CD4^+^T cells with a CAR construct and *FOXP3*, resulting in functional, stable, and suppressive Tregs in vitro. While these CAR-Tregs exhibited antigen-specific proliferation and preserved a Treg phenotype. Unfortunately, their infusion did not prevent diabetes in NOD/LtJ mice. However, the Tregs survived for approximately four months in vivo and had minimal off-target toxicity, highlighting their potential for targeted therapy [117]. Further optimization and targeting of additional antigens in β-cells may be needed for clinical efficacy.

Gene therapy advances allowed physicians to turn the abnormal CD4^+^ T cells into Treg-like cells by inserting a healthy *FOXP3* gene using lentiviral vectors. These engineered cells show the ability to suppress immune responses, both in lab studies and in humanized mouse models [118]. Likewise, genetically engineered hematopoietic stem cells carrying a healthy *FOXP3* gene could offer a potential cure [119]. The use of CRISPR/Cas9 enables accurate insertion of a healthy *FOXP3* gene [120]. T cells that constitutively express *FOXP3* following lentiviral transduction are being investigated in a Phase 1 clinical trial. This represents the first-in-human experimental therapy for immune dysregulation (NCT05241444) aiming to restore immune tolerance by functionally replacing defective Tregs in IPEX patients with engineered autologous T cells expressing *FOXP3* [121].

IL-2 signaling is essential for the maintenance, stability, and function of FOXP3^+^ Tregs. Studies in NOD mice showed that problems in the IL-2/CD25 pathway weaken Treg function. This can drive the development of autoimmune diabetes, pointing to IL-2 as both a key regulator and a potential treatment target [122]. Phase I/II trial tested ultra-low-dose IL-2 in 24 children (7–14 years) with recent type 1 diabetes in a multicenter, double-blind, placebo-controlled design. Patients received placebo or IL-2 (0.125, 0.250, 0.500 MIU/m^2^) daily for 5 days, then fortnightly for 1 year. Primary outcome was Treg change at day 5. IL-2 was safe, with only mild-to-moderate transient adverse events. Tregs increased dose-dependently, with seven high responders showing better 1-year C-peptide maintenance. Glycemic control was unchanged overall. IL-2 safely expands Tregs, and high responders demonstrate preserved insulin production [123]. In a recent meta-analysis, IL-2 therapy achieved dose-dependent Treg expansion, ranging from 45% to 77% [124]. 

## 13. Future Directions and Challenges

Despite progress in understanding immune tolerance, the exact factors that disrupt *FOXP3* isoform regulation in endocrine autoimmune diseases remain poorly defined. A better understanding of how genetic predisposition, environmental influences, and post-transcriptional mechanisms interact are needed to clarify the causes of this dysregulation.

A key limitation in linking *FOXP3* to endocrine autoimmune diseases is that not all affected individuals show reduced frequencies of FOXP3^+^ T cells. Several studies have found that Treg numbers may remain normal, but their suppressive function is impaired. This suggests that FOXP3/Treg abnormalities are often qualitative. This reflects defects in Treg stability or functional capacity rather than numerical deficiency.

Despite that *FOXP3* plays an important role in autoimmune endocrinopathies, it is not the only determinant of the disease. Interactions with other genes and environmental factors are important and determine disease severity [65]. Therapies aimed at *FOXP3*; whether by boosting Treg numbers, using gene therapy, or adjusting gene expression, hold promise for restoring immune balance and helping patients. Nevertheless, further research is needed to fully study this potential.

## Data Availability

No new data were created or analyzed in this study. Data sharing is not applicable to this article.

## Supplementary Materials

The following supporting information can be downloaded at: https://www.mdpi.com/article/10.3390/ijms27135778/s1.
