# Supplementary material for: FOXP3 Mutations and Instability as Determinants of Regulatory T-Cell Plasticity in Endocrine Autoimmunity
Source: Int J Mol Sci. 2026 Jun 26;27(13):5778. doi: 10.3390/ijms27135778 (PMC13362172; doi:10.3390/ijms27135778)
Supplement: Supplementary file 1 [file ijms-27-05778-s001.zip › ijms-4337591-supplementary.pdf]

Table S1: Thirty two pathogenic mutations in the *FOXP3* gene in IPEX syndrome. Source ClinVar (https://www.ncbi.nlm.nih.gov/clinvar).

Variation	Gene (Protein Change)	Type (Consequence)	Condition
NM_014009.4(FOXP3):c.1290_*12delinsTG (p.Pro431fs)	FOXP3 (P396fs +1 more)	Indel (frameshift variant)	IPEX
NM_014009.4(FOXP3):c.1293_1294del (p.Ter432ThrextTer?)	FOXP3	Deletion (frameshift variant)	IPEX
NM_014009.4(FOXP3):c.1234del (p.Glu412fs)	FOXP3 (E377fs +1 more)	Deletion (frameshift variant)	IPEX
NM_014009.4(FOXP3):c.1222G>A (p.Val408Met)	FOXP3 (V408M +1 more)	Single nucleotide variant (missense variant)	FOXP3-related disorder (+1 more)
NM_014009.4(FOXP3):c.1190G>A (p.Arg397Gln)	FOXP3 (R397Q +1 more)	Single nucleotide variant (missense variant)	Not provided (+1 more)
NM_014009.4(FOXP3):c.1189C>T (p.Arg397Trp)	FOXP3 (R397W +1 more)	Single nucleotide variant (missense variant)	Hydrops fetalis (+1 more)
NM_014009.4(FOXP3):c.1150G>A (p.Ala384Thr)	FOXP3 (A384T +1 more)	Single nucleotide variant (missense variant)	IPEX
NM_014009.4(FOXP3):c.1117_1118delinsGC (p.Phe373Ala)	FOXP3 (F373A +1 more)	Indel (missense variant)	IPEX
NM_014009.4(FOXP3):c.1112T>G (p.Phe371Cys)	FOXP3 (F371C +1 more)	Single nucleotide variant (missense variant)	IPEX
NM_014009.4(FOXP3):c.1110G>A (p.Met370Ile)	FOXP3 (M335I +1 more)	Single nucleotide variant (missense variant)	IPEX
NM_014009.4(FOXP3):c.1099T>C (p.Phe367Leu)	FOXP3 (F367L +1 more)	Single nucleotide variant (missense variant)	IPEX
NM_014009.4(FOXP3):c.1087A>G (p.Ile363Val)	FOXP3 (I328V +1 more)	Single nucleotide variant (missense variant)	IPEX
NM_014009.4(FOXP3):c.1040G>A (p.Arg347His)	FOXP3 (R312H +1 more)	Single nucleotide variant (missense variant)	IPEX (+1 more)
NM_014009.4(FOXP3):c.1015C>G (p.Pro339Ala)	FOXP3 (P339A +1 more)	Single nucleotide variant (missense variant)	Not provided (+1 more)
NM_014009.4(FOXP3):c.1010G>A (p.Arg337Gln)	FOXP3 (R302Q +1 more)	Single nucleotide variant (missense variant)	Not provided (+2 more)
NM_014009.4(FOXP3):c.906del (p.Asp303fs)	FOXP3 (D303fs +1 more)	Deletion (frameshift variant)	IPEX
NM_014009.4(FOXP3):c.751_753del (p.Glu251del)	FOXP3 (E216del +1 more)	Deletion (inframe_deletion)	Not provided (+1 more)
NM_014009.4(FOXP3):c.748_750del (p.Lys250del)	FOXP3 (K250del +1 more)	Deletion (inframe_deletion)	IPEX (+1 more)

NM_014009.4(FOXP3):c.736-2A>T	FOXP3	Single nucleotide variant (splice acceptor variant)	IPEX
NM_014009.4(FOXP3):c.727del (p.Glu243fs)	FOXP3 (E208fs +1 more)	Deletion (frameshift variant)	IPEX
NM_014009.4(FOXP3):c.694T>G (p.Cys232Gly)	FOXP3 (C232G +1 more)	Single nucleotide variant (missense variant)	IPEX
NM_014009.4(FOXP3):c.227del (p.Leu76fs)	FOXP3 (L76fs)	Deletion (frameshift variant +1 more)	IPEX
NM_014009.4(FOXP3):c.224C>T (p.Pro75Leu)	FOXP3 (P75L)	Single nucleotide variant (missense variant +1 more)	IPEX
NM_014009.4(FOXP3):c.210+1G>A	FOXP3	Single nucleotide variant (splice donor variant)	IPEX
NM_014009.4(FOXP3):c.210+1G>T	FOXP3	Single nucleotide variant (splice donor variant)	IPEX
NM_014009.4(FOXP3):c.210+1G>C	FOXP3	Single nucleotide variant (splice donor variant)	Not provided
NM_014009.4(FOXP3):c.142C>T (p.Arg48Ter)	FOXP3 (R48*)	Single nucleotide variant (nonsense)	IPEX
NM_014009.4(FOXP3):c.3G>A (p.Met1Ile)	FOXP3 (M1I)	Single nucleotide variant (missense variant +1 more)	IPEX
NM_014009.4(FOXP3):c.2T>A (p.Met1Lys)	FOXP3 (M1K)	Single nucleotide variant (missense variant +1 more)	IPEX
NM_014009.4(FOXP3):c.-23+1G>T	FOXP3	Single nucleotide variant (splice donor variant)	IPEX
NC_000023.10:g.(?*46466387)*(50659607_?)del	AKAP4, ARAF (+89 more)	Deletion	IPEX
FOXP3, 543C>T	FOXP3	Single nucleotide variant	IPEX
